# Associations between Response to Commonly Used Neo-Adjuvant Schedules in Rectal Cancer and Routinely Collected Clinical and Imaging Parameters

**DOI:** 10.3390/cancers14246238

**Published:** 2022-12-18

**Authors:** Masoud Karimi, Pia Osterlund, Klara Hammarström, Israa Imam, Jan-Erik Frodin, Bengt Glimelius

**Affiliations:** 1 Department of Gastrointestinal Oncology, Karolinska University Hospital, 171 64 Stockholm, Sweden; 2 Department of Oncology and Pathology, Karolinska Institutet, 171 77 Stockholm, Sweden; 3 Department of Oncology, Tampere University Hospital, University of Tampere, 33520 Tampere, Finland; 4 Department of Immunology, Genetics and Pathology, Uppsala University, 752 36 Uppsala, Sweden

**Keywords:** rectal cancer, predictive factors, pre-operative treatment, pathologic complete remission

## Abstract

**Simple Summary:**

We studied real-world patients with locally advanced rectal cancer receiving preoperative radiotherapy with or without chemotherapy. The aim was to find factors associated with complete response to therapy, i.e., no remaining tumour, that could be used to identify patients who would not need surgery in the future. Tumour stage and length, intensity of preoperative treatment, and laboratory factors, such as carcinoembryonic antigen (CEA), leucocyte counts, and platelets, were all associated with complete response. Treatment intensity mattered and when radiotherapy was combined with chemotherapy, 21% had a complete response compared to 8% with radiotherapy alone. A model for identifying patients with a better chance of achieving a complete response was developed using tumour stage and length, CEA, and leukocyte levels as factors predicting complete response.

**Abstract:**

Complete pathological response (pCR) is achieved in 10–20% of rectal cancers when treated with short-course radiotherapy (scRT) or long-course chemoradiotherapy (CRT) and in 28% with total neoadjuvant therapy (scRT/CRT + CTX). pCR is associated with better outcomes and a “watch-and-wait” strategy (W&W). The aim of this study was to identify baseline clinical or imaging factors predicting pCR. All patients with preoperative treatment and delays to surgery in Uppsala-Dalarna (*n* = 359) and Stockholm (*n* = 635) were included. Comparison of pCR versus non-pCR was performed with binary logistic regression models. Receiver operating characteristics (ROC) models for predicting pCR were built using factors with *p* < 0.10 in multivariate analyses. A pCR was achieved in 12% of the 994 patients (scRT 8% [33/435], CRT 13% [48/358], scRT/CRT + CTX 21% [43/201]). In univariate and multivariate analyses, choice of CRT (OR 2.62; 95%CI 1.34–5.14, scRT reference) or scRT/CRT + CTX (4.70; 2.23–9.93), cT1–2 (3.37; 1.30–8.78; cT4 reference), tumour length ≤ 3.5 cm (2.27; 1.24–4.18), and CEA ≤ 5 µg/L (1.73; 1.04–2.90) demonstrated significant associations with achievement of pCR. Age < 70 years, time from radiotherapy to surgery > 11 weeks, leucocytes ≤ 10^9^/L, and thrombocytes ≤ 400^9^/L were significant only in univariate analyses. The associations were not fundamentally different between treatments. A model including T-stage, tumour length, CEA, and leucocytes (with scores of 0, 0.5, or 1 for each factor, maximum 4 points) showed an area under the curve (AUC) of 0.66 (95%CI 0.60–0.71) for all patients, and 0.65–0.73 for the three treatments separately. The choice of neoadjuvant treatment in combination with low CEA, short tumour length, low cT-stage, and normal leucocytes provide support in predicting pCR and, thus, could offer guidance for selecting patients for organ preservation.

## 1. Introduction

Administration of preoperative radiotherapy, either short-course (scRT) or long-course, has been important in decreasing local recurrence rates after rectal cancer surgery [1,2,3,4,5]. In locally advanced rectal cancer (LARC), the addition of chemotherapy to long-course radiotherapy (CRT) further decreases local recurrence rates, whereas the impact on overall survival (OS), except possibly in the most advanced (ugly) cases, is unclear [6,7,8]. An impact on distant metastasis-free survival rates has only been seen when preoperative chemotherapy (CTX) is added to scRT/CRT, i.e., total neoadjuvant therapy (TNT) [9,10].

The tumour response to neoadjuvant treatment is highly heterogenous, ranging from complete shrinkage of the tumour to lack of effect and, occasionally, to progression. In approximately 10–28% of patients, neoadjuvant treatment results in the disappearance of the rectal tumour with no signs of residual tumour on MRI, proctoscopy, and digital examination (clinical complete response, cCR) or no residual viable cancer cells in the surgical specimen and pathologic complete response (pCR, also denoted ypCR) [9,10,11]. Patients who achieve pCR exhibit significantly more favourable oncological outcomes with higher 5-year disease-free survival rates (DFS, 83% if pCR versus 66% if non-pCR) and OS rates (88% with pCR versus 76% non-pCR) [12,13]. The achievement of a complete remission provides the possibility for organ-preserving surgery or a non-operative, watch-and-wait (W&W) approach. Research efforts have been directed toward identification of clinical and other parameters that could help predict pCR after preoperative treatment [14,15,16,17,18,19]. However, several unclear issues, particularly related to the importance of clinical T-stage (cT-stage), tumour length, presence of extramural vascular invasion (cEMVI+), mesorectal fascia (cMRF+) involvement, and clinical laboratory values need to be clarified [14,15,16,17,18,19].

The primary aim of this study was to identify clinical and imaging factors that can be used to predict pCR (and would thus also be applicable to cCR with the possibility of a W&W strategy), by combining data from two population-based Swedish cohorts, and comparing the three most commonly used schedules, scRT, CRT, or scRT/CRT + CTX; all with a ≥4-week interval to surgery. A secondary aim was to explore if a model based on clinical factors predicting pCR could be built.

## 2. Materials and Methods

Our study consisted of two independent population-based retrospectively collected cohorts from Uppsala-Dalarna (Cohort A) and Stockholm (Cohort B). All patients living in these regions at the time of diagnosis constituted the study base and were included if they had received preoperative RT with or without chemotherapy with a minimum delay of 4 weeks to surgery. Patients in Cohort A were treated between 1 January 2010 and 31 December 2018; a detailed description of this cohort has been published previously [15]. Cohort B consisted of data for consecutive patients with rectal cancer in the Stockholm region diagnosed between 1 January 2006 and 31 December 2016. Patients were followed for recurrence and survival until 24 March 2022. Several patients in both cohorts were included in randomized trials such as Stockholm III (42 in Cohort A and 54 in Cohort B), EXPERT-C (0/33), or RAPIDO (106/35) [9,20,21].

The results of cohort A have been published [15]; we initially sought to determine whether the two patient datasets demonstrated similar associations between the explored variables and pCR; if so, they could be combined to increase statistical power in the calculations of importance for, above all, the three different treatment schedules most commonly used today.

The study was approved by the local ethical committee at Karolinska Institutet as an extension to the approval by the ethical committee at Uppsala University for the Uppsala/Dalarna study. Through approval, we gained access to the prospectively maintained quality register database including all patients in these regions diagnosed with colorectal cancer. This database is part of the national quality register (Swedish Colorectal Cancer Registry [SCRCR] defined in detail at https://scrcr.se/) (accessed on 7 November 2022).

The same inclusion criteria were applied for both cohorts, except that, patients who achieved cCR (*n* = 22) and were monitored in a W&W strategy were included in the Uppsala/Dalarna study. However, in this analysis, the cCR patients were excluded to achieve histopathological data from the resected specimen for all patients. The differences in collected data between the cohorts included the presence of metastatic lateral lymph nodes according to MRI and thrombocytes collected only in Cohort B. Of the total 361 cases in cohort A, two patients were inadvertently missed before fusion with Cohort B, leaving 359 patients from cohort A for final analysis. Patients with rectal cancer and M0 disease treated with curative intent who received preoperative neoadjuvant or conversion treatment (hereafter referred to as preoperative) followed by delayed surgery performed ≥4 weeks after completion of the RT were eligible. This study excluded patients with concurrent malignant disease and crucial missing information about pathological staging.

Rectal tumours were defined as those located with the caudal limit within 15 cm above the anal verge, the distance being measured by rigid proctoscopy. Low rectal tumours were defined as those 0–5 cm above the anal verge, middle rectum as 6–10 cm, and high tumours as more than 10 cm above the anal verge. The database contains information about clinical, radiological, and histopathological staging, information on all treatments, relapse sites and timepoints, and survival information. This information was retrieved from the SCRCR and the patient’s medical files. Cut-off ≤ 3.5 cm for tumour length was defined with the ROC/Youden method (area under curve [AUC] 0.55; 95% CI 0.50–0.61, *p =* 0.050). The other cut-offs were defined as previously published [22,23,24].

For both cohorts, neoadjuvant treatment was given according to one of three different protocols:

A: scRT: short-course hypo-fractionated 5 Gy × 5 in one week, and delayed surgery.

B: CRT: Chemoradiation 1.8 Gy × 28 or 2 × 25 Gy concomitant with capecitabine 825 mg/m^2^ twice daily, days 1–38 or 900 mg/m^2^ on RT days, and delayed surgery.

C: scRT/CRT + CTX: neoadjuvant scRT or CRT preceded or followed by chemotherapy as part of the clinical trials EXPERT-C and RAPIDO. The EXPERT-C randomised phase II trial administered four cycles of capecitabine and oxaliplatin (CAPOX) alone or with cetuximab, followed by CRT, TME-surgery, and further four cycles of adjuvant CAPOX [21]. The RAPIDO study compared CRT as standard arm versus an experimental arm starting with 5 Gy × 5, followed by six courses of CAPOX before surgery [9]. Adjuvant chemotherapy using 8 cycles of CAPOX was provided in the standard arm.

Radiology included pelvic MRI and chest, abdominal, and pelvic CT at baseline used as a basis in these analyses. Restaging 3–6 weeks after completion of the neoadjuvant treatment served as grounds for decision making regarding curative surgery during a multidisciplinary team (MDT) conference, but these results were not included in the analyses.

### Statistics

Data were analysed with SPSS (IBM SPSS Statistics for Windows, 2020, Version 27.0.0.1, IBM Corporation, Armonk, NY, USA). Patient demographics are presented as absolute values and percentages, and, for continuous variables, also as median and range. Comparisons of groups were performed by applying the Χ^2^ test for categorical variables. For continuous variables, we applied the non-parametric Mann–Whitney U test or the Wilcoxon signed rank test. Laboratory tests (haemoglobin, leucocytes, thrombocytes, C-reactive protein, and CEA) were analysed as categorical factors. With pCR as a dependent parameter, we used models of binary logistic regression for univariate analyses and calculated odds ratios (OR) and 95% confidence intervals (CI) to predict whether covariates influenced the achievement of pCR. Variables that were associated with pCR in the univariate analyses with *p* < 0.05 for all patients and <0.10 for treatment groups, and with missing values less than 18% were included in the multivariate logistic regression analyses. Receiver operating characteristics (ROC) with Youden optimization and AUC were calculated to discriminate for the model’s predictive power. The cut-offs for the factors from the multivariate analysis added to the model were the ones used in the Sorbye consensus [22] and those optimized by the Youden method as described above. Relapse-free survival (RFS), overall survival (OS), and disease-specific survival (DSS) were calculated with Kaplan–Meier survival estimates and the Cox proportional hazards model. All *p*-values were two-sided and considered statistically significant when *p* < 0.05.

## 3. Results

### 3.1. Patient Characteristics

Patient and tumour characteristics for the 359 patients in Cohort A and the 635 in Cohort B are described in Table S1. Minor differences in age and baseline MRI-derived factors, such as cN-stage, cMRF+, cEMVI+ and mucinous tumour, as well as clinical factors such as elevated carcinoembryonic antigen (CEA) distribution were noted. The proportion of patients treated with the three alternatives, scRT, CRT, or scRT/CRT + CTX, varied between the cohorts because of the differences in inclusion times, with Cohort A formed in 2010–2018, and Cohort B in 2006–2016. Prior to June 2011, when the RAPIDO trial comparing CRT and scRT + CTX was initiated, most LARCs were treated with CRT and after June 2016, and when the trial had closed patient entry, these patients continued to be treated with scRT + CTX within the LARCTC-US study (https://clinicaltrials.gov/ct2/show/NCT03729687) (accessed on 23 November 2022). Different treatment schedules resulted in varying times to surgery. Patient and tumour characteristics in the treatment groups (scRT, CRT, and scRT/CR + CTX) in the two cohorts were also in line.

When the associations between the characteristics and the probability of reaching pCR (12.8% in Cohort A and 12.3% in Cohort B) were compared between the cohorts, similar results were observed (Table S2). Because of this, we concluded that the findings reported from Cohort A [15] were confirmed in an independent cohort (Cohort B) and the two cohorts could be pooled.

The clinical characteristics of the pooled cohort (A + B) by treatment group are described in Table 1. Patients treated with scRT were older and had less advanced tumours (cT1-3, cN0, cMRF-, or cEMVI-) according to treatment indication, and had fewer mucinous tumours and shorter tumour lengths, but higher C-reactive protein (CRP) levels. The most advanced tumours were observed in the scRT/CRT + CTX group.

Survival was compared between the pCR and non-pCR groups. The median reverse Kaplan–Meier follow-up was at 64 months (95% CI 63–65). RFS was significantly better in the pCR group (HR 0.22; 95% CI 0.13–0.37), with 5-year RFS rates of 96% in pCR versus 79% in non-pCR groups (Figure S1A). OS was better in the pCR group with a 5-year OS rate of 92% compared with 70% in the non-pCR group (Figure S1B). DSS, considering CRC deaths and censoring deaths from other causes, was also higher in the pCR arm, with a 5-year DSS rate of 96% in the pCR group versus 79% in the non-pCR group (Figure S1C).

### 3.2. Clinical Factors and pCR

pCR was achieved in 12% of the 994 patients. pCR was noted in 8% (33/435) with scRT, in 13% (48/358) with CRT, and in 21% (43/201) with scRT/CRT + CTX (*p* < 0.001).

Characteristics of the patients who achieved pCR compared to those who did not are presented in Table 2. Tumour characteristics such as cT-stage (*p =* 0.027) and tumour length (*p =* 0.010) were statistically significantly associated with pCR. Furthermore, laboratory parameters, including leucocytosis (*p* = 0.014), thrombocytosis (*p =* 0.023), elevated CRP (*p* < 0.001), and CEA (*p =* 0.001) were statistically significantly different between the pCR and non-pCR groups. The cohort did not show any difference.

Data regarding the probability of reaching pCR according to treatment are shown in Table 3. Significant differences for all patients were noted for age, cT-stage, cN-stage, cMRF, cEMVI, mucinous tumour, and weeks from RT to surgery for treatment (exact *p*-values not shown). Differences in pCR rates for the scRT group were noted for cT-stage, cMRF, cEMVI, and CEA. For the CRT group, differences in pCR versus non-pCR were noted for cT-stage, tumour length, and elevated CEA. For the scRT/CRT + CTX arm, only sex was statistically significant in the pCR versus non-pCR comparison.

### 3.3. Univariate and Multivariate Analyses for pCR

Patients achieving pCR were compared to non-pCR patients for clinical and tumour-related factors (Table 4). In the univariate binary logistic regression analyses for pCR, statistical significance was noted for age ≤ 70 years (OR 2.09, 95% CIs in Table 4), cT1-2 (OR 2.47, with T4 as reference), tumour length ≤3.5 cm (OR 1.84), time from RT to surgery (OR 1.57), normal leucocytes (OR 2.37), normal thrombocytes (OR 4.57), normal CEA (OR 2.03), or CRT (OR 1.89, with scRT as reference), and scRT/CRT + CTX (OR 3.32 with scRT as reference).

In multivariate analysis (*n* = 735 with 98 events), seven covariates with *p*-value < 0.05 in the univariate analyses were included, excluding thrombocytes not collected in Cohort A. Factors that were statistically significant for pCR in the multivariate model included cT1-2 (OR 3.37 with cT4 as reference), tumour length ≤ 3.5 cm (OR 2.27), non-elevated CEA (OR 1.73), and CRT (OR 2.61 with scRT as reference) or scRT/CRT + CTX (OR 4.70 with scRT as reference). Interaction terms for the significant factors were not significant and thus not included in the multivariate model (*p*-value for cT-stage + tumour length was 0.963, cT-stage + CEA was 0.957, and cT-stage + treatment group was 0.850).

We examined predictive factors associated with pCR for the three treatments separately. In the scRT group (*n* = 435), univariate analyses demonstrated that age ≤ 70 years, cT1-2, cMRF-, cEMVI-, and normal thrombocytes were associated with higher pCR rates (Table S3), with none of the factors remaining statistically significant in the multivariate analysis (*n* = 294). In the CRT population (*n* = 358), cT1-2, tumour length ≤ 3.5 cm, and normal CEA were associated with a higher pCR rate in the univariate analyses, and cT1-2 (OR 5.94) remained statistically significant in the multivariate analysis (*n* = 301, Table S4). In the scRT/CRT + CTX group (*n* = 201), female sex with OR 2.00 was the only statistically significant factor in the univariate analyses (Table S5).

### 3.4. Predictive Model for pCR

A predictive model for pCR was developed based on factors identified in the multivariable analysis (Table S6, Figure S2). Cut-offs were based on TNM classification, the literature, and the ROC defined.

The scoring points for cT-stage (cT1-2 = 0, cT3 = 0.5, and cT4 = 1), tumour length (≤3.5 cm = 0, 4–7 cm = 0.5, and >7 cm = 1), CEA (≤3 µg/L = 0, 3–5 µg/L = 0.5, and >5 µg/L = 1), and leucocytes (≤8.2–10^9^/L = 0, 8.3–10^9^/L = 0.5, and >10^9^/L = 1) were combined, thus resulting in a maximum score of 4. ORs and 95% CIs are presented in Table S6.

The performance of the combined pCR effects model obtained an AUC of 0.65 (95% CI 0.60–0.71), with cut-off < 1.75 points for the whole cohort (*p* < 0.001), of which 25% had pCR. In the subgroup treated with scRT, AUC was 0.73 (0.62–0.83) and cut-off < 1.25 points and 16% had pCR; in the CRT group, AUC was 0.67 (0.58–0.76) with cut-off < 1.75 points and 31% had pCR; and for scRT/CRT + CTX, AUC was 0.65 (0.55–0.75) with cut-off < 1.75 points and 50% had pCR (all statistically significant).

## 4. Discussion

The highest pCR rates of 21% were achieved with scRT/CRT + CTX compared with 13% with CRT and 8% with scRT in this pooled analysis of rectal cancer patients who underwent surgery after a delay following pre-treatment. Independent factors associated with pCR were cT1-2, tumour length ≤ 3.5 cm, normal CEA, and treatment modality. Leucocytosis also adds to the model. This may have practical importance when discussing whether a non-surgical W&W approach could be recommended prior to treatment initiation.

Our population-based results indicate that treatment with RT, either preceded or followed by systemic chemotherapy, i.e., TNT, is the most effective treatment modality for achieving pCR. In recent years, the focus has been directed towards more extensive administration of chemotherapy in the neoadjuvant setting and several clinical trials have been performed or are ongoing [9,10,25,26]. Results from the RAPIDO study demonstrated the superiority of scRT + CTX versus CRT in preventing disease-related events, predominantly systemic recurrences, and support our findings regarding the hierarchy of preoperative treatments in achieving pCR [9]. Despite a two-fold higher chance of pCR in the experimental group (28% versus 14%) [9], a 5-year update of the trial has revealed more locoregional failures in the experimental group (12% vs. 8%, *p* = 0.07) [27]. Results from the US OPRA study with CRT and chemotherapy either as induction or consolidation showed better organ preservation rates (3-year TME-free survival rate 41% vs. 53%), when the chemotherapy was given as consolidation after CRT [11].

Several studies have reported cT-stage as an independent variable to predict pCR [28,29], in line with our findings. Our cohort of patients with cT1-2 tumours was limited (*n* = 62, as most patients with cT1-2 tumours underwent surgery directly or were treated with scRT without a delay to surgery and were thus excluded from analysis), but achieved a pCR rate of 23%, and this was as high as 50% in the CRT group. With a corresponding rate for cT3 tumours of 13% we can conclude that, not surprisingly, cT1-2-stage can be used as predictive factor for pCR when using scRT or CRT. Most cT2-stage patients are regularly not candidates for pre-surgical treatment, and too few patients with cT1-2 tumours were treated with scRT/CRT + CTX to draw any conclusions. In Cohort A, a subdivision of cT3-stage into the substages a-d was explored [15]; however, this was not recorded in cohort B. It is possible that the best discriminator is not between cT2 and cT3 but rather within cT3. Separation of cT2 from cT3a is also difficult using MRI [30].

Length of tumour persistently demonstrated statistically significant associations with pCR in our examination for the entire cohort. We found a cut-off of ≤3.5 cm to be a break point for tumours responding with pCR. The calculated AUC 0.55 indicates, however, a limited discriminative strength and necessitates incorporation of other parameters when predicting pCR. A study by Jankowski et al. [31] showed that a tumour length > 7 cm and circumferent extension of the tumour meant that only 1.6% could achieve a sustained cCR with a sensitivity as low as 23% [31]. In our study, we did not retrieve data for the extent of circumferential tumour engagement of the rectal wall; thus, our results are not comparable with the Polish study. It is fully plausible that cT-stage and tumour length overlap as larger tumours are more often associated, but not necessarily always (reflected in non-significant interaction term), with higher cT-stage with deeper invasion into the rectal wall. In this way, their significance as predictive factors may intertwine.

The serum marker CEA has been used to predict prognosis both pre- and post-operatively, and it is an important tool for surveillance of colorectal patients to detect recurrence post-operatively [24]. CEA has also been the subject of interest as a predictor for response to neo-adjuvant therapy in rectal cancer, most studies of which have found associations with pCR rates [14,15,28,32,33,34]. In a report from Joye et al., a CEA cut-off of 4.6 µg/L was applied and an association between pre-treatment CEA and probability for pCR was found in a multivariable analysis [14]. Furthermore, in our study, a pre-treatment CEA value below reference (≤5 µg/L) had a positive association with an OR of 1.28 for reaching pCR (whereas the ROC-defined subgroup with ≤3 µg/L had an OR of 2.39). CEA was the only laboratory parameter in the full cohort that demonstrated statistically significant associations with pCR in both univariate and multivariable analyses; however, the number of patients with missing values for the other laboratory tests was quite high (9–45%).

The significance of age in univariate analyses is probably related to the active selection of younger fit patients for more intense treatment. Older patients, often with comorbidities, may not always tolerate these treatments and are left with scRT alone, which has less cell killing effect and, thus, fewer pCRs, as seen in a systematic review [35].

Our findings of both leucocytosis and thrombocytosis being significant covariate factors in univariate analyses are in accordance with previous reports in LARC [17,36,37,38,39]. These associations were most pronounced in the scRT/CRT + CTX-group. Thrombocytosis could not be added to the multivariate model as this information was available only in Cohort B. Pre-treatment haemoglobin value and its relation to oncologic treatment response (particularly RT) and prognosis in solid tumours, including rectal cancer, have been the focus of several studies [14,28,40]. A higher pre-treatment haemoglobin value is associated with pCR likelihood [14,28], in line with a trend in our study. Clinically, haemoglobin values are probably of limited relevance.

MRI-defined cMRF+ and cEMVI+ were significantly associated with pCR in the scRT group but not in the CRT or scRT/CRT + CTX groups. Both involved MRF and positive EMVI indicate a more advanced tumour and the reference treatment is either CRT or scRT/CRT + CTX. Thus, scRT was provided only to fragile patients not tolerating the reference treatment. Therefore, if a suboptimal treatment must be given, with scRT for an advanced tumour, both MRF+ and EMVI+ mean a lower chance of pCR. If the reference treatment is applied, neither of these factors are important for predicting pCR (or potentially for a cCR if a W&W policy is applied).

In summary, besides treatment protocol, early tumour stage (cT1-2), tumour length ≤ 3.5 cm, normal routine blood counts, and CEA can assist in predicting pCR and, ultimately, cCR state in a setting before neoadjuvant treatment initiation. The decision to aim for organ preservation by giving more active neoadjuvant treatment than indicated can thus be supported by our model, with the caveat of its limitation as AUCs were in the range of 0.65–0.73, sensitivity and specificity 59–70%, and pCR carried rates of 16–50%. The predictive factors and the model also need to be validated in other large patient series, preferably prospective, and we will start with the RAPIDO [9] dataset. In this regard, other markers, serial examinations with MRI, PET-CT, and functional radiology measuring the tumour’s metabolic activity before and early during the treatment could improve the baseline model in the future [41].

Better survival has also been observed in patients achieving pCR [12,13] in line with our findings for OS, DSS, and RFS (with 5-year rates of 92%, 96%, and 92%, respectively). pCR status thus helps in decisions to omit adjuvant therapy [42]. A third decision our prognostic factors may support is to sustain from surgery in cCR and offer a W&W strategy. Today, this is normally based on tumour-free proctoscopy, digital examination, and MRI. Still, in this situation, there is a clear risk that tumour cells persist [43]. It has been reported that a near-pCR situation is not associated with the same favourable prognosis as pCR [44,45]. This adds to the dilemma and necessitates incorporation of further tools to judge durable tumour control probability with better certainty.

Our study has limitations associated with retrospective studies. The exclusion of cCR patients also reduced the number of favourable outcome patients in cohort A. Undeniably, there has been a selection bias as many patients were selected for different treatment protocols based on age and comorbidities. In terms of strengths, this was a comprehensive study that included a large number of patients treated with the three most widely utilized neoadjuvant protocols after up-to-date staging, including an MRI for all patients.

## 5. Conclusions

The choice of neoadjuvant treatment in combination with low CEA, short tumour length, low cT-stage, and normal leucocytes provide support in predicting pCR and, thus, could offer guidance for selection of patients for organ preservation strategies at baseline, i.e., to provide neoadjuvant rather than adjuvant treatments and W&W strategies.

## Figures and Tables

**Table 1:** Baseline clinical, imaging, and laboratory characteristics of the pooled cohort of eligible patients (*n* = 994) according to pre-operative treatment.

Treatment		scRT	CRT	scRT/CRT + CTX	All Patients	*p*-Value
		*n* = 435 (44%)	*n* = 358 (36%)	*n* = 201 (20%)	*n* = 994 (100%)	
Age	Median (range)	73 (43–91)	64 (31–81)	64 (23–82)	66 (23–91)	**0.003**
	≤70 years	183 (42%)	287 (80%)	156 (78%)	626(63%)	**<0.001**
	>70 years	252 (58%)	71 (20%)	45 (22%)	368 (37%)	
Sex	Female	173 (40%)	150 (42%)	85 (42%)	408 (41%)	0.768
	Male	262 (60%)	208 (58%)	116 (58%)	586 (59%)	
MRI T-stage	cT1-2	50 (12%)	10 (3%)	2 (1%)	62 (6%)	**<0.001**
	cT3	260 (60%)	170 (47%)	102 (51%)	532 (54%)	
	cT4	125 (28%)	178 (50%)	96 (48%)	399 (40%)	
	Missing	0 (0%)	0 (0%)	1 (0.5%)	1 (0.1%)	
MRI N-stage	cN0	124 (29%)	37 (10%)	10 (5%)	171 (17%)	**<0.001**
	cN1-2	309 (71%)	321 (90%)	191 (95%)	821 (83%)	
	Missing	2 (0.5%)	0 (0%)	0 (0%)	2 (0.2%)	
MRI Mesorectal	No	254 (58%)	86 (24%)	61 (30%)	401 (40%)	**<0.001**
fascia engagement	Yes	175 (40%)	272 (76%)	139 (69%)	586 (59%)	
	Missing	6 (2%)	0 (0%)	1 (0.5%)	7 (0.7%)	
MRI Extramural	No	296 (68%)	201 (56%)	94 (47%)	591 (60%)	**<0.001**
vascular invasion	Yes	129 (30%)	157 (44%)	105 (52%)	391 (39%)	
	Missing	10 (2%)	0 (0%)	2 (1%)	12 (1%)	
MRI Mucinous tumour	No	369 (85%)	301 (84%)	142 (71%)	812 (82%)	**<0.001**
	Yes	55 (13%)	57 (16%)	56 (28%)	168 (17%)	
	Missing	11 (2%)	0 (0%)	3 (1%)	14 (1%)	
MRI Lateral lymph nodes	No	242 (56%)	202 (56%)	52 (26%)	496 (50%)	**<0.001**
	Yes	44 (10%)	68 (19%)	27 (13%)	139 (14%)	
	Missing	149 (34%)	88 (25%)	122 (61%)	359 (36%)	
MRI Tumour length	≤3.5 cm	87 (20%)	27 (8%)	22 (11%)	136 (14%)	**<0.001**
	>3.5 cm	320 (74%)	319 (89%)	173 (86%)	812 (82%)	
	Missing	27 (6%)	12 (3%)	6 (3%)	45 (4%)	
Distance anal verge	0–5 cm	179 (41%)	144 (40%)	64 (32%)	387 (39%)	0.050
	6–10 cm	164 (38%)	147 (41%)	80 (40%)	391 (39%)	
	11–15 cm	92 (21%)	67 (19%)	57 (28%)	216 (22%)	
Weeks from end of RT	≤8	244 (56%)	141 (39%)	29 (14%)	414 (42%)	**<0.001**
to surgery	8–11	92 (21%)	133 (37%)	18 (9%)	243 (24%)	
	>11	99 (23%)	84 (24%)	154 (77%)	337 (34%)	
Haemoglobin	>110 g/L	291 (67%)	327 (91%)	179 (89%)	797 (80%)	0.100
	≤110 g/L	54 (12%)	30 (8%)	21 (10%)	105 (11%)	
	Missing	90 (21%)	1 (0.2%)	1 (0.5%)	92 (9%)	
Leucocytes	≤10^9^/L	257 (59%)	302 (84%)	155 (77%)	714 (72%)	0.122
	>10^9^/L	63 (15%)	49 (14%)	28 (14%)	140 (14%)	
	Missing	115 (26%)	7 (2%)	18 (9%)	140 (14%)	
Thrombocytes	≤400^9^/L	176 (41%)	239 (67%)	69 (34%)	484 (49%)	0.598
	>400^9^/L	19 (4%)	31 (9%)	10 (5%)	60 (6%)	
	Missing	240 (55%)	88 (24%)	122 (61%)	450 (45%)	
C-reactive protein	≤10 mg/L	186 (43%)	251 (70%)	108 (54%)	545 (55%)	**<0.001**
	>10 mg/L	86 (20%)	47 (13%)	45 (22%)	178 (18%)	
	Missing	163 (38%)	60 (17%)	48 (24%)	271 (27%)	
Carcinoembryonic antigen	≤5 µ/L	198 (45%)	187 (52%)	119 (59%)	504 (51%)	0.441
	>5 µ/L	107 (25%)	124 (35%)	76 (38%)	307 (31%)	
	Missing	130 (30%)	47 (13%)	6 (3%)	183 (18%)	
Pathologic	Non-pCR	402 (92%)	310 (87%)	158 (79%)	870 (87%)	**<0.001**
complete response	pCR	33 (8%)	48 (13%)	43 (21%)	124 (13%)	

Abbreviations: CRT: concomitant chemoradiotherapy, MRI: magnetic resonance imaging, pCR: pathologic complete response, RT: radiotherapy, scRT: short course radiotherapy, scRT/CRT + CTX: scRT/CRT combined with systemic chemotherapy, MRI Tumour length: craniocaudal extension of tumour measured by MRI. p-values below 0.05 are marked in bold.

**Table 2:** Differences in baseline clinical, laboratory, and imaging-defined characteristics and treatment groups between the pCR and the non-pCR groups for the pooled cohort (*n* = 994).

		Non-pCR	pCR	*p*-Value
		*n* = 870 (Row%)	*n* = 124 (Row%)	
Age	Median (range)	68 (23–91)	65 (38–84)	**0.003**
	≤70 years	531 (85%)	95 (15%)	**0.001**
	>70 years	339 (92%)	29 (8%)	
Sex	Female	351 (86%)	57 (14%)	0.234
	Male	519 (89%)	67 (11%)	
MRI T-stage	cT1-2	48 (77%)	14 (23%)	**0.027**
	cT3	464 (87%)	68 (13%)	
	cT4	357 (90%)	42 (10%)	
	Missing	1	0	
MRI N-stage	cN0	152 (89%)	19 (11%)	0.546
	cN1-2	716 (87%)	105 (13%)	
	Missing	2	0	
MRI Mesorectal fascia	No	346 (86%)	55 (14%)	0.366
engagement	Yes	517 (88%)	69 (12%)	
	Missing	7	0	
MRI Extramural	No	513 (87%)	78 (13%)	0.190
vascular invasion	Yes	348 (89%)	43 (11%)	
	Missing	9	3	
MRI Mucinous tumour	No	713 (88%)	99 (12%)	0.456
	Yes	144 (86%)	24 (14%)	
	Missing	13	1	
MRI Lateral lymph nodes	No	441 (89%)	55 (11%)	0.083
	Yes	116 (84%)	23 (17%)	
	Missing	313	46	
MRI Tumour length	≤3.5 cm	109 (80%)	27 (20%)	**0.010**
	>3.5 cm	716 (88%)	96 (12%)	
	Missing	44	1	
Distance anal verge	0–5 cm	332 (86%)	55 (14%)	0.414
	6–10 cm	347 (89%)	44 (11%)	
	11–15 cm	191 (88%)	25 (12%)	
Weeks from end of RT	≤8	371 (90%)	43 (10%)	0.110
to surgery	8–11	214 (88%)	29 (12%)	
	>11	285 (85%)	52 (15%)	
Haemoglobin	>110 g/L	689 (86%)	108 (14%)	0.088
	≤110 g/L	97 (92%)	8 (8%)	
	Missing	84	8	
Leucocytes	≤10^9^/L	614 (86%)	100 (14%)	**0.014**
	>10^9^/L	131 (94%)	9 (6%)	
	Missing	125	15	
Thrombocytes	≤400^9^/L	418 (86%)	66 (14%)	**0.023**
	>400^9^/L	58 (97%)	2 (2%)	
	Missing	394	56	
C-reactive protein	≤10 mg/L	468 (86%)	77 (14%)	**<0.001**
	>10 mg/L	160 (90%)	18 (10%)	
	Missing	242	29	
Carcinoembryonic antigen	≤5 µ/L	424 (84%)	80 (16%)	**0.001**
	>5 µ/L	281 (92%)	26 (8%)	
	Missing	165	18	
Treatment group	scRT	402 (92%)	33 (8%)	**<0.001**
	CRT	310 (87%)	48 (13%)	
	scRT/CRT + CTX	158 (79%)	43 (21%)	
Cohort	A Uppsala/Dalarna	313 (87%)	46 (13%)	0.808
	B Stockholm	557 (88%)	78 (12%)	

Abbreviations: CRT: concomitant chemoradiotherapy, MRI: magnetic resonance imaging, pCR: pathologic complete response, RT: radiotherapy, scRT: short course radiotherapy, scRT/CRT + CTX: scRT/CRT combined with systemic chemotherapy, MRI Tumour length: craniocaudal extension of tumour measured by MRI. p-values below 0.05 are marked in bold.

**Table 3:** Observed frequencies of pCR according to major clinical parameters in the treatment groups.

		scRT			CRT			scRT/CRT + Chemo			Total		
		Total	pCR		Total	pCR		Total	pCR		Total	pCR	
		435	33	Row %	358	48	Row %	201	43	Row %	994	124	Row %
Sex	Female	173	10	6%	150	23	15%	85	24	28%	408	57	14%
	Male	262	23	9%	208	25	12%	116	19	16%	586	67	11%
Age	≤70 years	183	19	10%	287	42	15%	156	34	22%	626	95	15%
	>70 years	252	14	6%	71	6	8%	45	9	20%	368	29	8%
MRI T-stage	cT1-2	50	9	18%	10	5	50%	2	0	0%	62	14	23%
	cT3	260	19	7%	170	23	14%	102	26	25%	532	68	13%
	cT4	125	5	4%	178	20	11%	96	17	18%	399	42	11%
	Missing	0	0		0	0		1	0	0%	1	0	0%
MRI N-stage	cN0	124	12	10%	37	5	14%	10	2	20%	171	19	11%
	cN1-2	309	21	7%	321	43	13%	191	41	21%	821	105	13%
	Missing	2	0	0%	0	0		0	0		2	0	0%
MRI Mesorectal fascia	MRF-	254	26	10%	86	14	16%	61	15	25%	401	55	14%
	MRF+	175	7	4%	272	34	13%	139	28	20%	586	69	12%
	Missing	6	0	0%	0	0		1	0	0%	7	0	0%
MRI Extramural	EMVI-	296	28	9%	201	30	15%	94	20	21%	591	78	13%
vascular invasion	EMVI+	129	4	3%	157	18	11%	105	21	20%	391	43	11%
	Missing	10	1	10%	0	0		2	2	100%	12	3	25%
MRI Mucinous tumour	Non-mucinous	369	30	8%	301	40	13%	142	29	20%	812	99	12%
	Mucinous	55	2	4%	57	8	14%	56	14	25%	168	24	14%
	Missing	11	1	9%	0	0		3	0	0%	14	1	7%
MRI Lateral lymph	No lat. nodes	242	18	7%	202	26	13%	52	11	21%	496	55	11%
nodes	Lateral nodes	44	5	11%	68	11	16%	27	7	26%	139	23	17%
	Missing	149	10	7%	88	11	13%	122	25	20%	359	46	13%
MRI Tumour length	≤3.5 cm	87	10	11%	27	9	33%	22	8	36%	136	27	20%
	>3.5 cm	320	23	7%	319	38	12%	173	35	20%	812	96	12%
	Missing	27	0	0%	12	1	8%	6	0	0%	45	1	2%
Distance anal verge	0–5 cm	179	16	9%	144	22	15%	64	17	27%	387	55	14%
	6–10 cm	164	10	6%	147	17	12%	80	17	21%	391	44	11%
	11–15 cm	92	7	8%	67	9	13%	57	9	16%	216	25	12%
Weeks from RT to	≤8	244	22	9%	141	17	12%	29	4	14%	414	43	10%
surgery	8–11	92	7	8%	133	20	15%	18	2	11%	243	29	12%
	>11	99	4	4%	84	11	13%	154	37	24%	337	52	15%
Haemoglobin	>110 g/L	291	22	8%	327	47	14%	179	39	22%	797	108	14%
	≤110 g/L	54	3	6%	30	1	3%	21	4	19%	105	8	8%
	Missing	90	8	9%	1	0	0%	1	0	0%	92	8	9%
Leucocytes	≤10^9^/L	257	19	7%	302	44	15%	155	37	24%	714	100	14%
	>10^9^/L	63	3	5%	49	3	6%	28	3	11%	140	9	6%
	Missing	115	11	10%	7	1	14%	18	3	17%	140	15	11%
Thrombocytes	≤400^9^/L	176	13	7%	239	35	15%	69	18	26%	484	66	14%
	>400^9^/L	19	0	0%	31	2	6%	10	0	0%	60	2	3%
	Missing	240	20	8%	88	11	13%	122	25	20%	450	56	12%
C-reactive protein	≤10 mg/L	186	14	8%	251	36	14%	108	27	25%	545	77	14%
	>10 mg/L	86	4	5%	47	5	11%	45	9	20%	178	18	10%
	Missing	163	15	9%	60	7	12%	48	7	15%	271	29	11%
Carcinoembryonic	≤5 µ/L	198	18	9%	187	32	17%	119	30	25%	504	80	16%
antigen	>5 µ/L	107	3	3%	124	11	9%	76	12	16%	307	26	8%
	Missing	130	12	9%	47	5	11%	6	1	17%	183	18	10%

Abbreviations: CRT: concomitant chemoradiotherapy, MRI: magnetic resonance imaging, pCR: pathologic complete response, RT: radiotherapy, scRT: short course radiotherapy, scRT/CRT + CTX: scRT/CRT combined with systemic chemotherapy, MRI Tumour length: craniocaudal extension of tumour measured by MRI. Differences that are statistically significant (X^2^-test) in the different treatments are marked in bold.

**Table 4:** Univariate and multivariate logistic regression analyses of the pooled cohort (*n* = 994 and 735, respectively) for clinical, laboratory, and imaging-defined factors predicting pCR status.

		Univariate Analyses *n* = 994		Multivariable Model *n* = 735	
		OR (95% CI)	*p*-Value	OR (95% CI)	*p*-Value
Age	Continuous	0.97 (0.95–0.99)	**0.002**		
	>70 years	1.00		1.00	
	≤70 years	2.09 (1.35–3.23)	**0.001**	1.35 (0.77–2.37)	0.291
Sex	Male	1.00			
	Female	1.25 (0.86–1.83)	0.234		
MRI T-stage	cT4	1.00		1.00	
	cT3	1.24 (0.82–1.87)	0.292	1.38 (0.85–2.28)	0.193
	cT1-2	2.47 (1.26–4.87)	**0.008**	3.37 (1.30–8.78)	**0.013**
MRI N-stage	cN1-2	1.00			
	cN0	1.173 (0.70–1.97)	0.546		
MRI Mesorectal fascia	MRF+	1.00			
engagement	MRF-	1.19 (0.81–1.74)	0.367		
MRI Extramural vascular	EMVI+	1.00			
invasion	EMVI-	1.23 (0.82–1.82)	0.305		
MRI Mucinous tumour	Mucinous	1.00			
	Non-mucinous	1.20 (0.72–1.94)	0.456		
MRI Lateral lymph nodes	Lateral lymph nodes	1.00			
	No lateral lymph nodes	0.62 (0.37–1.06	0.085		
MRI Tumour length	>3.5 cm	1.00		1.00	
	≤3.5 cm	1.84 (1.15–2.96)	**0.011**	2.27 (1.24–4.18)	**0.008**
Distance anal verge	0–5 cm	1.00			
	6–10 cm	0.76 (0.50–1.17)	0.217		
	11–15 cm	0.79 (0.47–1.30)	0.154		
Weeks from RT to Surg.	≤8	1.00		1.00	
	8–11	1.16 (0.70–1.92)	0.540	1.61 (0.87–2.98)	0.131
	>11	1.57 (1.02–2.42)	**0.040**	1.45 (0.79–2.67)	0.227
Haemoglobin	≤110 g/L	1.00			
	>110 g/L	1.90 (0.89–4.01)	0.093		
Leucocytes	>10^9^/L	1.00		1.00	
	≤10^9^/L	2.37 (1.16–4.81)	**0.017**	2.02 (0.93–4.37)	0.075
Thrombocytes	>400^9^/L	1.00			
	≤400^9^/L	4.57 (1.09–19.2)	**0.037**		
C-reactive protein	≤10 mg/L	1.00			
	>10 mg/L	1.46 (0.85–2.52)	0.171		
Carcinoembryonic antigen	>5 µ/L	1.00		1.00	
	≤5 µ/L	2.03 (1.27–3.25)	**0.003**	1.73 (1.04–2.90)	**0.034**
Treatment group	scRT	1.00		1.00	
	CRT	1.89 (1.18–3.01)	**0.008**	2.621 (1.34–5.14)	**0.005**
	scRT/CRT + CTX	3.32 (2.03–5.41)	**<0.001**	4.70 (2.23–9.93)	**<0.001**

Abbreviations: CRT: concomitant chemoradiotherapy, MRI: magnetic resonance imaging, pCR: pathologic complete response, RT: radiotherapy, scRT: short course radiotherapy, scRT/CRT + CTX: scRT/CRT combined with systemic chemotherapy, MRI Tumour length: craniocaudal extension of tumour measured by MRI. p-values below 0.05 are marked in bold.

## Data Availability

Data can be made available by written request to the corresponding author.

## Supplementary Materials

The following supporting information can be downloaded at: https://www.mdpi.com/article/10.3390/cancers14246238/s1, Table S1: Comparison of major clinical and imaging characteristics of cohort A and cohort B; Table S2: Comparison of clinical, laboratory, and imaging-defined characteristics between pCR vs. non-pCR groups for cohort A (n = 359) and cohort B (n = 635); Table S3: Univariate and multivariate analyses of the scRT cohort (n = 435) for clinical, laboratory, and imaging-defined factors predicting pCR status; Table S4: Univariate and multivariate analyses of the CRT cohort (n = 358) for clinical, laboratory, and imaging-defined factors predicting pCR status; Table S5: Univariate and multivariate analyses of the scRT/CRT + CTX cohort (n = 201) for clinical, laboratory, and imaging-defined factors predicting pCR status; Table S6: Score board for the predictive pCR model; Figure S1: Relapse-free survival (RFS; panel A), overall survival (OS; panel B), and disease-specific survival (DSS) of patients with tumours achieving a pathologic complete response (pCR) compared to non-pCR; Figure S2: Receiver operator characteristics (ROC) and area under the curve (AUC) with cut-offs optimized by Youden for the model including MRI cT-stage, tumour length, elevated CEA, and leucocytosis for all patients (ALL; panel A), short-course radiotherapy (scRT; panel B), chemoradiation (CRT, panel C), and scRT/CRT aS2: and chemotherapy (scRT/CRT + CTX, panel D) groups.
