# Supplementary material for: Associations between Response to Commonly Used Neo-Adjuvant Schedules in Rectal Cancer and Routinely Collected Clinical and Imaging Parameters
Source: Cancers (Basel). 2022 Dec 18;14(24):6238. doi: 10.3390/cancers14246238 (PMC9777013; doi:10.3390/cancers14246238)

Table S1. **Comparison of major clinical and imaging characteristics of cohort A (Uppsala/Dalarna regions) and cohort B (Stockholm region)**

		Cohort A n=359 (36%)	Cohort B n=635 (64%)	P-Value
Age	Median (range)	68 (31-91)	66 (23-89)	**0.003**
	≤ 70 years	209 (58%)	417 (66%)	**0.019**
	> 70 years	150 (42%)	218 (34%)	
Sex	Female	153 (43%)	255 (40%)	0.449
	Male	206 (57%)	380 (60%)	
MRI T-stage	cT1-2	16 (5%)	46 (7%)	0.165
	cT3	201 (56%)	331 (52%)	
	cT4	141 (39%)	258 (41%)	
	Missing	1	0	
MRI N-stage	cN0	45 (13%)	126 (20%)	**0.003**
	cN1-2	313 (87%)	508 (80%)	
	Missing	1	1	
MRI Mesorectal fascia engagement	No	165 (46%)	236 (37%)	**0.003**
	Yes	187 (52%)	399 (63%)	
	Missing	7	0	
MRI Extramural Vascular invasion	No	192 (54%)	399 (63%)	**0.022**
	Yes	155 (43%)	236 (37%)	
	Missing	12 (3%)	0	
MRI Mucinous tumour	No	238 (66%)	574 (90%)	**0.001**
	Yes	107 (30%)	61 (10%)	
	Missing	14 (4%)	0	
MRI Lateral lymph nodes	No	0	496 (78%)	NA
	Yes	0	139 (22%)	
	Missing	359 (100)	0	
MRI Tumour length	≤3.5 cm	52 (15%)	84 (13%)	0.816
	>3.5cm	302 (84%)	510 (80%)	
	Missing	5	40 (6%)	
Distance anal verge	0-5 cm	137 (38%)	250 (39%)	0.434
	6-10 cm	136 (38%)	255 (40%)	
	11-15 cm	86 (24%)	130 (21%)	
Weeks from end of RT to surgery	≤8	115 (32%)	299 (47%)	**<0.001**
	8-11	86 (24%)	157 (25%)	
	>11	158 (44%)	179 (28%)	
Haemoglobin	>110 g/L	306 (85%)	491 (77%)	0.209
	≤110 g/L	47 (13%)	58 (9%)	
	Missing	6	86 (14%)	
Leucocytes	≤10 ^9/L	261 (72%)	453 (72%)	0.727
	>10 ^9/L	49 (14%)	91 (14%)	
	Missing	49 (14%)	91 (14%)	
Thrombocytes	≤400 ^9/L	0	484 (76%)	NA
	>400 ^9/L	0	60 (9%)	
	Missing	359 (100%)	91 (14%)	
C-reactive protein	≤10 mg/L	222 (62%)	323 (51%)	0.550
	>10 mg/L	68 (19%)	110 (17%)	
	Missing	69	202	
Carcinoembryonic antigen	≤5 µ/L	188 (52%)	316 (50%)	**0.007**
	>5 µ/L	144 (40%)	163 (26%)	
	Missing	27 (8%)	156 (25%)	
Treatment group	scRT	149 (42%)	286 (45%)	**<0.001**
	CRT	88 (25%)	270 (43%)	
	scRT/CRT + CTX	122 (34%)	79 (12%)	
Pathologic complete response	Non-pCR	313 (87%)	557 (88%)	0.808
	pCR	46 (13%)	78 (12%)	

Abbreviations: CRT: *concomitant chemoradiotherapy*, **MRI:** Magnetic resonance imaging, **pCR:** *Pathologic complete response*, **RT:** *Radiotherapy*, **scRT:** *short course radiotherapy*, **scRT/CRT+CTX:** *scRT/CRT combined with systemic chemotherapy,* **MRI Tumour length***: craniocaudal extension of tumour measured by MRI.*

Table S2. Comparison of clinical, laboratory and imaging defined characteristics between pCR vs non-pCR group for cohort A (n=359) and cohort B (n=635)

		Cohort A			Cohort B		
		**Non-pCR n=313 (Row %)**	**pCR n=46 (Row %)**	**P-value**	**Non-pCR n=557 (Row %)**	**pCR n=78 (Row %)**	**P-value**
Age	Median (range)	69 (31-91)	66 (41-84)	***0.010***	67 (23-89)	65 (38-83)	**0.040**
	≤ 70 years	173 (83%)	36 (17%)	***0.003***	358 (86%)	59 (14%)	**0.048**
	> 70 years	140 (93%)	10 (7%)		199 (91%)	19 (9%)	
Sex	Female	129 (84%)	24 (16%)	0.160	222 (87%)	33 (13%)	0.679
	Male	184(89%)	22 (11%)		335 (88%)	45 (12%)	
MRI T-stage	cT1-2	13 (81%)	3 (19%)	***0.031***	35 (76%)	11 (24%)	0.656
	cT3	174 (87%)	27 (13%)		290 (88%)	41 (12%)	
	cT4	125 (89%)	16 (11%)		232 (90%)	26 (10%)	
	Missing	1	0		0	0	
MRI N-stage	cN0	41 (91%)	4 (9%)	0.396	111 (88%)	15 (12%)	0.879
	cN1-2	271 (87%)	42 (13%)		445 (88%)	63 (12%)	
	Missing	1	0		1	0	
MRI Mesorectal fascia engagement	No	141 (86%)	24 (14%)	0.440	205 (87%)	31 (13%)	0.615
	Yes	165 (88%)	22 (12%)		352 (88%)	47 (12%)	
	Missing	7	0		0	0	
MRI Extramural vascular invasion	No	168 (88%)	24 (12%)	0.946	345 (86%)	54 (14%)	0.212
	Yes	136 (88%)	19 (12%)		212 (90%)	24 (10%)	
	Missing	9	3		0	0	
MRI Mucinous tumour	No	207 (87%)	31 (13%)	0.988	506 (88%)	68 (12%)	0.304
	Yes	93 (87%)	14 (13%)		51 (84%)	10 (16%)	
	Missing	13 (93%)	1		0	0	
MRI Lateral lymph nodes	No	0	0	NA	441 (89%)	55 (11%)	0.083
	Yes	0	0		116 (84%)	23 (17%)	
	Missing	313	46		0	0	
MRI Tumour length	≤3.5 cm	42 (81%)	10 (19%)	0.148	67 (80%)	17 (20%)	**0.032**
	>3.5cm	266 (88%)	36 (12%)		450 (88%)	60 (12%)	
	Missing	5	0		39 (98%)	1	
Distance anal verge	0-5 cm	117 (85%)	20 (15%)	0.197	215 (86%)	35 (14%)	0.292
	6-10 cm	124 (91%)	12 (9%)		223 (87%)	32 (13%)	
	11-15 cm	72 (84%)	14 (16%)		119 (91%)	11 (9%)	
Weeks from end of RT to surgery	≤8	108 (94%)	7 (6%)	***0.025***	263 (88%)	36 (12%)	0.672
	8-11	74 (86%)	12 (14%)		140 (89%)	17 (11%)	
	>11	131 (83%)	27 (17%)		154 (86%)	25 (14%)	
Haemoglobin	>110 g/L	265 (87%)	41 (13%)	0.601	424 (86%)	67 (14%)	0.067
	≤110 g/L	42 (89%)	5 (11%)		55 (95%)	3 (5%)	
	Missing	6	0		78 (91%)	8 (9%)	
Leucocytes	≤10 ^9/L	224 (86%)	37 (14%)	0.123	390 (86%)	63 (14%)	0.056
	>10 ^9/L	46 (94%)	3 (6%)		85 (93%)	6 (7%)	
	Missing	43 (88%)	6 (12%)		82 (90%)	9 (10%)	
Thrombocytes	≤400 ^9/L	0	0	NA	418 (86%)	66 (14%)	**0.023**
	>400 ^9/L	0	0		58 (97%)	2 (3%)	
	Missing	313	46		81	10	
C-reactive protein	≤10 mg/L	189 (85%)	33 (15%)	0.201	279 (86%)	44 (14%)	0.464
	>10 mg/L	62 (91%)	6 (9%)		98 (89%)	12 (11%)	
	Missing	62	7		180	22	
Carcinoembryonic antigen	≤5 µ/L	158 (84%)	30 (16%)	0.097	266 (84%)	50 (16%)	**0.009**
	>5 µ/L	130 (90%)	14 (10%)		151 (93%)	12 (7%)	
	Missing	25 (93%)	2 (7%)		140 (90%)	16 (10%)	
Treatment group	scRT	139 (93%)	10 (7%)	***0.003***	263 (92%)	23 (8%)	**0.001**
	CRT	77 (88%)	11 (13%)		233 (86%)	37 (14%)	
	scRT/CRT +CTX	97 (80%)	25 (21%)		61 (77%)	18 (23%)	

Abbreviations: CRT: *concomitant chemoradiotherapy*, **MRI:** Magnetic resonance imaging, **pCR:** *Pathologic complete response*, **RT:** *Radiotherapy*, **scRT:** *short course radiotherapy*, **scRT/CRT+CTX:** *scRT/CRT combined with systemic chemotherapy,* **MRI Tumour length***: craniocaudal extension of tumour measured by MRI. P-values below 0.05 are marked in bold.*

Table S3. Uni- and multivariate analyses of the *scRT* cohort (n=435) for clinical, laboratory and imaging defined factors predicting pCR status

		**Univariate analysis n=435**		**Multivariable model n=294**	
Age	Continuous	0.96 (0.93-0.99)	**0.030**		
	> 70 years	1.00		1.00	
	≤ 70 years	1.97 (0.96-4.04)	0.064	1.75 (0.65-4.67)	0.266
Sex	Male	1.00			
	Female	0.64 (0.29-1.38)	0.251		
MRI T-stage	cT4	1.00			
	cT3	1.89 (0.69-5.19)	0.216	2.34 (0.33-16.7)	0.395
	cT1-2	5.27 (1.67-16.62	**0.005**	6.52 (0.70-60.6)	0.099
MRI N-stage	cN1-2	1.00			
	cN0	1.47 (0.70-3.90)	0.309		
MRI Mesorectal fascia	MRF+	1.00		1.00	
engagement	MRF-	2.74 (1.16-6.46)	**0.021**	1.06 (0.22-5.16)	0.940
MRI Extramural vascular	EMVI+	1.00		1.00	
invasion	EMVI-	3.27 (1.12-9.51)	**0.030**	1.89 (0.50-7.16)	0.350
MRI Mucinous tumour	Mucinous	1.00			
	Non-mucinous	2.35 (0.54-10.10)	0.253		
MRI Lateral lymph nodes	Lateral lymphnodes	1.00			
	No lateral lymphnodes	0.63 (0.22-1.79	0.382		
Tumour length	>3.5cm	1.00			
	≤3.5 cm	1.68 (0.77-3.67)	0.196		
Distance anal verge	0-5cm	1.00			
	6-10 cm	0.66 (0.29-1.50)	0.323		
	11-15cm	0.84 (0.33-2.12)	0.710		
Weeks from RT to Surg.	≤8	1.00			
	8-11	0.83 (0.34-2.02)	0.682		
	>11	0.43 (0.14-1.27)	0.125		
Haemoglobin	≤110 g/L	1.00			
	>110 g/L	1.39 (0.40-4.82)	0.603		
Leucocytes	>10 ^9/L	1.00			
	≤10 ^9/L	1.60 (0.46-5.57)	0.463		
Thrombocytes	>400 ^9/L	1.00			
	≤400 ^9/L	4.58 (1.09-19.2)	**0.037**		
C-reactive protein	≤10 mg/L	1.00			
	>10 mg/L	1.67 (0.53-5,23)	0.380		
Carcinoembryonic antigen	>5 µ/L	1.00		1.00	
	≤5 µ/L	4.58 (1.09-19.2)	0.050	1.97 (0.52-7.39)	0.315

Abbreviations: CRT: *concomitant chemoradiotherapy*, **MRI:** Magnetic resonance imaging, **pCR:** *Pathologic complete response*, **RT:** *Radiotherapy*, **scRT:** *short course radiotherapy*, **scRT/CRT+CTX:** *scRT/CRT combined with systemic chemotherapy,* **MRI Tumour length***: craniocaudal extension of tumour measured by MRI. P-values below 0.05 are marked in bold.*

Table S4. Uni- and multivariate analyses of the *CRT* cohort (n=358) for clinical, laboratory and imaging defined factors predicting pCR status

		Univariate analysis n=358		Multivariable model n=301	
Age	Continuous	0.98 (0.96-1.02)	0.341		
	> 70 years	1.00			
	≤ 70 years	1.86 (0.76-4.56	0.177		
Sex	Male	1.00			
	Female	1.33 (0.72-2.44)	0.365		
MRI T-stage	cT4	1.00		1.00	
	cT3	1.24 (0.65-2.34)	0.516	1.26 (0.62-2.53)	0.525
	cT1-2	7.90 (2.10-29.7)	**0.002**	5.94 (1.20-29.3)	**0.029**
MRI N-stage	cN1-2	1.00			
	cN0	1.01 (0.37-2.73)	0.984		
MRI Mesorectal fascia	MRF+	1.00			
engagement	MRF-	1.36 (0.69-2.68)	0.371		
MRI Extramural vascular	EMVI+	1.00			
invasion	EMVI-	1.36 (0.72-2.53)	0.342		
MRI Mucinous tumour	Mucinous	1.00			
	Non-mucinous	0.94 (0.41-2.13)	0.880		
MRI Lateral lymph nodes	Lateral lymphnodes	1.00			
	No lateral lymphnodes	0.76 (0.36-1.65)	0.494		
Tumour length	>3.5cm	1.00		1.00	
	≤3.5 cm	3.70 (1.55-8.82)	**0.003**	1.65 (0.52-5.25)	0.400
Distance anal verge	0-5cm	1.00			
	6-10 cm	0.72 (0.37-1.43)	0.354		
	11-15 cm	0.86 (0.37-1.98)	0.725		
Weeks from RT to Surg.	≤8	1.00			
	8-11	1.29 (0.64-2.59)	0.471		
	>11	1.10 (0.49-2.48)	0.819		
Haemoglobin	≤110 g/L	1.00			
	>110 g/L	4.87 (0.65-4.01)	0.124		
Leucocytes	>10 ^9/L	1.00			
	≤10 ^9/L	2.61 (0.78-8.77)	0.120		
Thrombocytes	>400 ^9/L	1.00			
	≤400 ^9/L	2.49 (0.57-10.9)	0.227		
C-reactive protein	≤10 mg/L	1.00			
	>10 mg/L	1.41 (0.52-3.79)	0.500		
Carcinoembryonic antigen	>5 µ/L	1.00			
	≤5 µ/L	2.13 (1.03-4.39)	**0.043**	1.70 (0.80-3.61)	0.167

Abbreviations: CRT: *concomitant chemoradiotherapy*, **MRI:** Magnetic resonance imaging, **pCR:** *Pathologic complete response*, **RT:** *Radiotherapy*, **scRT:** *short course radiotherapy*, **scRT/CRT+CTX:** *scRT/CRT combined with systemic chemotherapy,* **MRI Tumour length***: craniocaudal extension of tumour measured by MRI. P-values below 0.05 are marked in bold.*

Table S5. Uni- and multivariate analyses of the *scRT/CRT+CTX* cohort (n=201) for clinical, laboratory and imaging defined factors predicting pCR status

		Univariate analysis n=201		Multivariable model n=195	
Age	Continuous	1.01 (0.98-1.04)	0.53		
	> 70 years	1.00			
	≤ 70 years	1.86 (0.76-4.56)	0.177		
Sex	Male	1.00		1.00	
	Female	2.00 (1.02-3.97)	**0.045**	1.97 (0.99-3.93)	0.055
MRI T-stage	cT4	1.00			
	cT3	1.59 (0.80-3.16)	0.186		
	cT1-2	NA	NA		
MRI N-stage	cN1-2	1.00			
	cN0	0.91 (0.18-4.47)	0.912		
MRI Mesorectal fascia engagement	MRF+	1.00			
	MRF-	1.29 (0.63-2.64)	0.482		
MRI Extramural vascular invasion	EMVI+	1.00			
	EMVI-	1.08 (0.54-2.15)	0.824		
MRI Mucinous tumour	Mucinous	1.00			
	Non-mucinous	0.77 (0.37-1.60)	0.482		
MRI Lateral lymph nodes	Lateral lymphnodes	1.00			
	No lateral lymphnodes	0.77 (0.26-2.27)	0.632		
Tumour length	>3.5cm	1.00		1.00	
	≤3.5 cm	2.25 (0.87-5.79)	0.092	2.08 (0.80-5.43)	0.133
Distance anal verge	0-5 cm	1.00			
	6-10 cm	0.75 (0.34-1.61)	0.456		
	11-15 cm	0.52 (0.21-1.28)	0.154		
Weeks from RT to Surg.	≤8	1.00			
	8-11	0.78 (0.13-4.77)	0.789		
	>11	1.98 (0.65-6.05)	0.232		
Haemoglobin	≤110 g/L	1.00			
	>110 g/L	1.18 (0.38-3.72)	0.773		
Leucocytes	>10 ^9/L	1.00			
	≤10 ^9/L	2.61 (0.75-9.15	0.133		
Thrombocytes	>400 ^9/L	1.00			
	≤400 ^9/L	2.49 (0.57-10.90)	0.227		
C-reactive protein	≤10 mg/L	1.00			
	>10 mg/L	1.33 (0.57-3.12)	0.507		
Carcinoembryonic antigen	>5 μ/L	1.00			
	≤5 μ/L	1.80 (0.86-3.78)	0.122		

Abbreviations: CRT: *concomitant chemoradiotherapy*, **MRI:** Magnetic resonance imaging, **pCR:** *Pathologic complete response*, **RT:** *Radiotherapy*, **scRT:** *short course radiotherapy*, **scRT/CRT+CTX:** *scRT/CRT combined with systemic chemotherapy,* **MRI Tumour length***: craniocaudal extension of tumour measured by MRI. P-values below 0.05 are marked in bold.*

Table S6. Score board for the predictive pCR model

		Points	Non-pCR n=869	Row %	pCR n=124	Row %	Univariate analyses n=811-993	*P*-value	Multivariate analysis n=735	*P*-value
Clinical T-stage	cT1-2	0.0	48	77 %	14	23 %	2.48 (1.26-4.87)	**0.008**	1.63 (0.67-3.95)	0.278
	cT3	0.5	464	87 %	68	13 %	1.25 (0.83-1.87)	0.292	1.09 (0.67-1.77)	0.723
	cT4	1.0	357	90 %	42	11 %	1.00		1.00	
MRI Tumour length	≤3.5 cm	0.0	109	80 %	27	20 %	3.09 (1.46-6.50)	**0.003**	3.15 (1.26-7.86)	0.114
	4-7 cm	0.5	579	87 %	85	13 %	1.83 (0.95-3.52)	0.071	1.87 (0.86-4.08)	0.115
	>7cm	1.0	137	93 %	11	7 %	1.00		1.00	
Leucocytosis	≤8 ^9/L	0.0	458	85 %	82	15 %	2.61 (1.27-5.23)	**0.009**	2.28 (1.06-4.94)	**0.036**
	8.1-10 ^9/L	0.5	156	90 %	18	10 %	1.68 (0.73-3.86)	0.223	1.51 (0.58-2.26)	0.367
	>10 ^9/L	1.0	131	94 %	9	6 %	1.00		1.00	
Carcinoembyonic antigen	≤3 µ/L	0.0	289	82 %	64	18 %	2.39 (1.47-3.89)	**<0.001**	1.85 (1.10-3.12)	**0.020**
	3-5 µ/L	0.5	135	89 %	16	11 %	1.28 (0.66-2.47)	0.459	1.15 (0.58-2.26)	
	>5 µ/L	1.0	281	92 %	26	9 %	1.00		1.00	

Abbreviations: pCR: *Pathologic complete response*, **MRI Tumour length***: craniocaudal extension of tumour measured by MRI. P-values below 0.05 are marked in bold.*

Figure S1. Relapse-free survival (RFS; panel A), overall survival (OS; panel B), and disease-specific survival (DSS) of patients with tumours achieving a pathologic complete response (pCR) compared to non-pCR

A

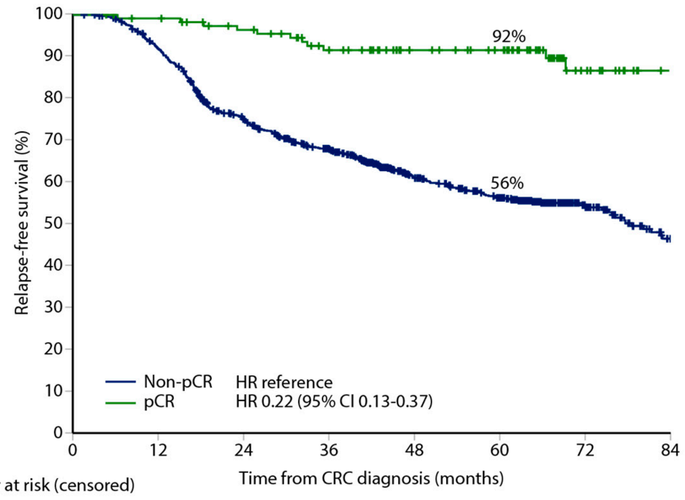

Number at risk (censored)

	0	12	24	36	48	60	72	84
Non-pCR	833 (12)	755 (45)	586 (81)	495 (207)	326 (238)	270 (398)	104 (433)	57 (466)
pCR	117 (3)	113 (9)	104 (17)	91 (33)	75 (40)	68 (80)	26 (93)	13 (103)

B

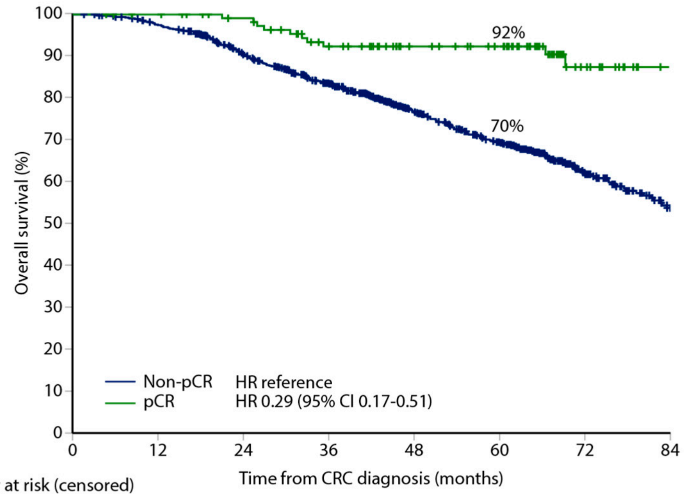

Number at risk (censored)

	0	12	24	36	48	60	72	84
Non-pCR	833 (12)	801 (51)	706 (96)	608 (234)	426 (273)	349 (452)	145 (495)	86 (545)
pCR	117 (3)	114 (9)	107 (17)	92 (33)	76 (40)	69 (81)	26 (94)	13 (104)

C

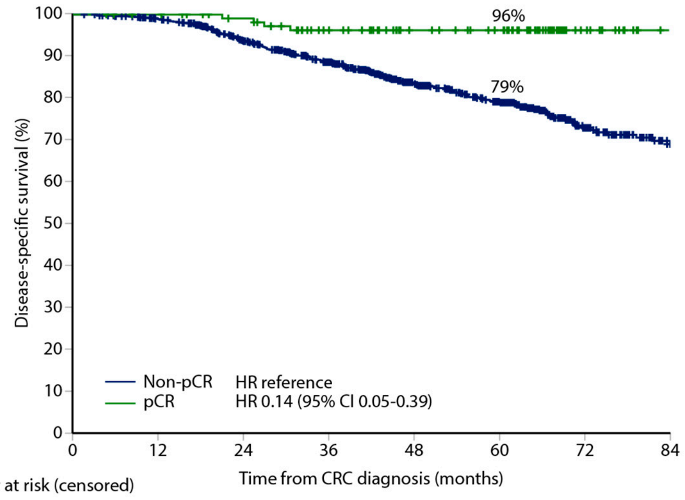

Number at risk (censored)

	0	12	24	36	48	60	72	84
Non-pCR	833 (24)	801 (78)	706 (139)	608 (290)	426 (347)	349 (533)	145 (586)	86 (658)
pCR	117 (3)	114 (9)	107 (21)	92 (37)	76 (44)	69 (87)	26 (100)	13 (113)

Figure S2. Receiver operator characteristics (ROC) and area under curve (AUC) with cut-offs optimized by Youden for the model including MRI cT-stage, tumour length, elevated CEA, and leucocytosis for all patients (ALL; panel A), short-course radiotherapy (scRT; panel B), chemoradiation (CRT, panel C), and scRT/CRT and chemotherapy (scRT/CRT + CTX, panel D) groups.

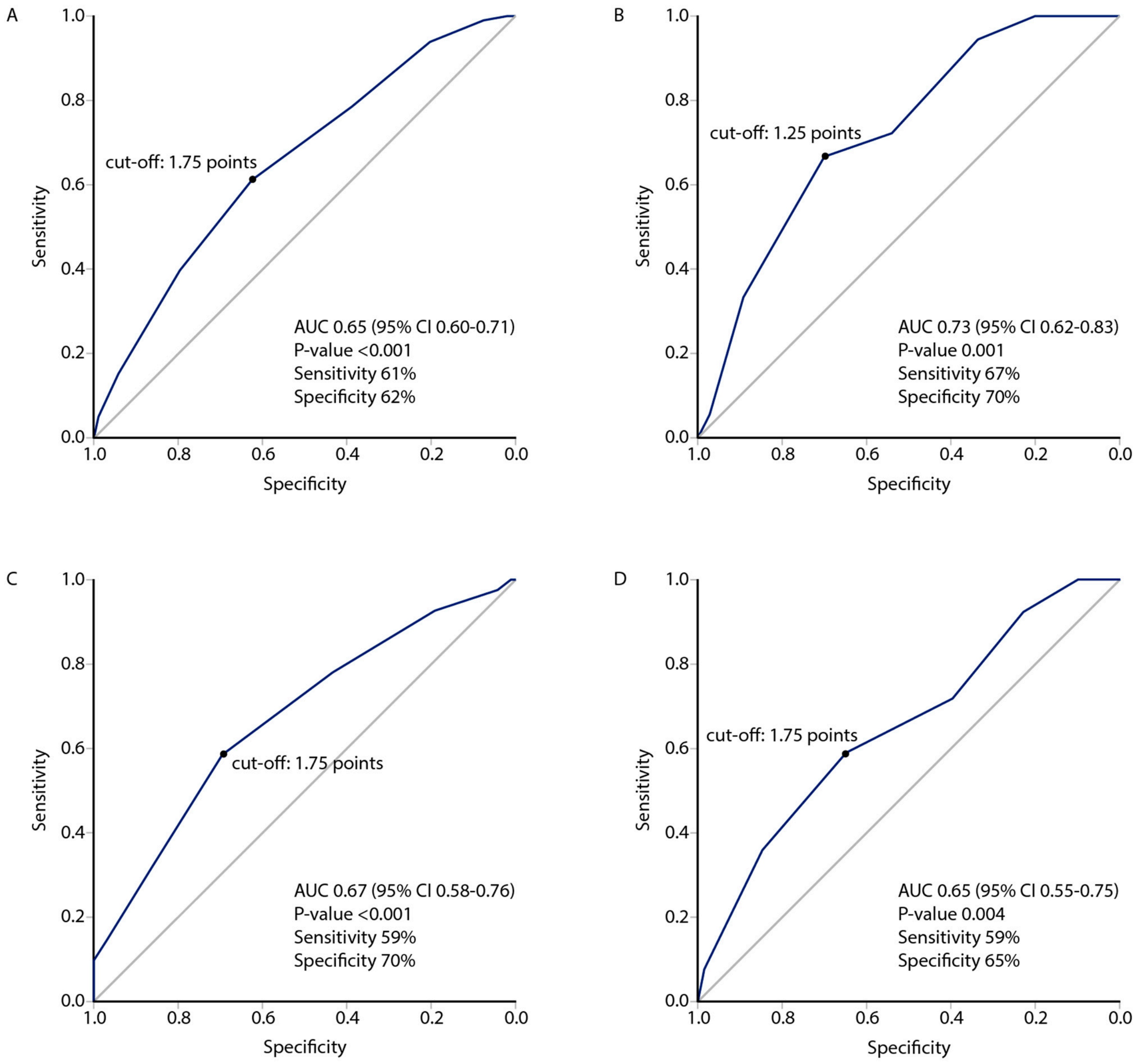

Supplement: Supplementary file 1 [file cancers-14-06238-s001.zip › cancers-2020295-supplementary.pdf]
